# Supplementary material for: Woodlice change the habitat use of spiders in a different food chain
Source: PeerJ. 2020 Jun 1;8:e9184. doi: 10.7717/peerj.9184 (PMC7271883; doi:10.7717/peerj.9184)
Supplement: Supplemental Information 1 [file peerj-08-9184-s001.docx]

**Supporting Information**

*Woodlice change the habitat use of spiders in a different food chain*

Stefanie M. Guiliano, Cerina M. Karr, Nathalie R. Sommer, and Robert W. Buchkowski

The following figures and table provide additional empirical data, detailed statistical results, an analysis of the wolf spider cages, and supplemental theoretical model results.

Interpreting the supporting information may be easier with the raw data and commented code files at <https://github.com/robertwbuchkowski/woodlice-and-nursery-web-spiders>.

**Section S1: Additional empirical results**

**
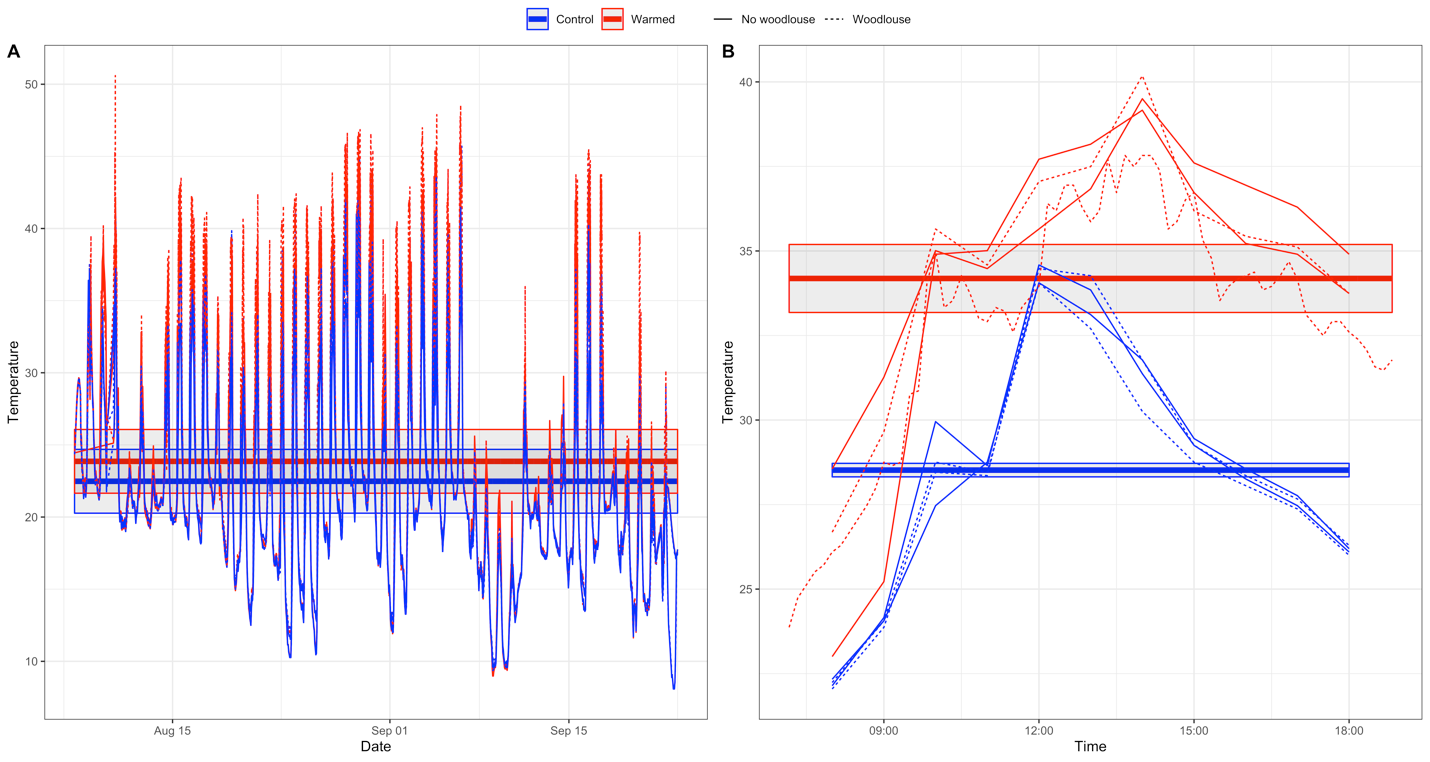
**

Figure S1: The temperature (°C) data recorded for control and warmed cages in 2018. The warmed cages were, on average 1°C warmed throughout the entire experiment when wrapped in plastic (A). During the behavioral trials on August 9/2018, the heat lamps raised the average cage temperature by 9°C (B). Thin lines show individual cages, while thick lines and shading show the average temperature ±1 standard deviation calculated at the cage level.


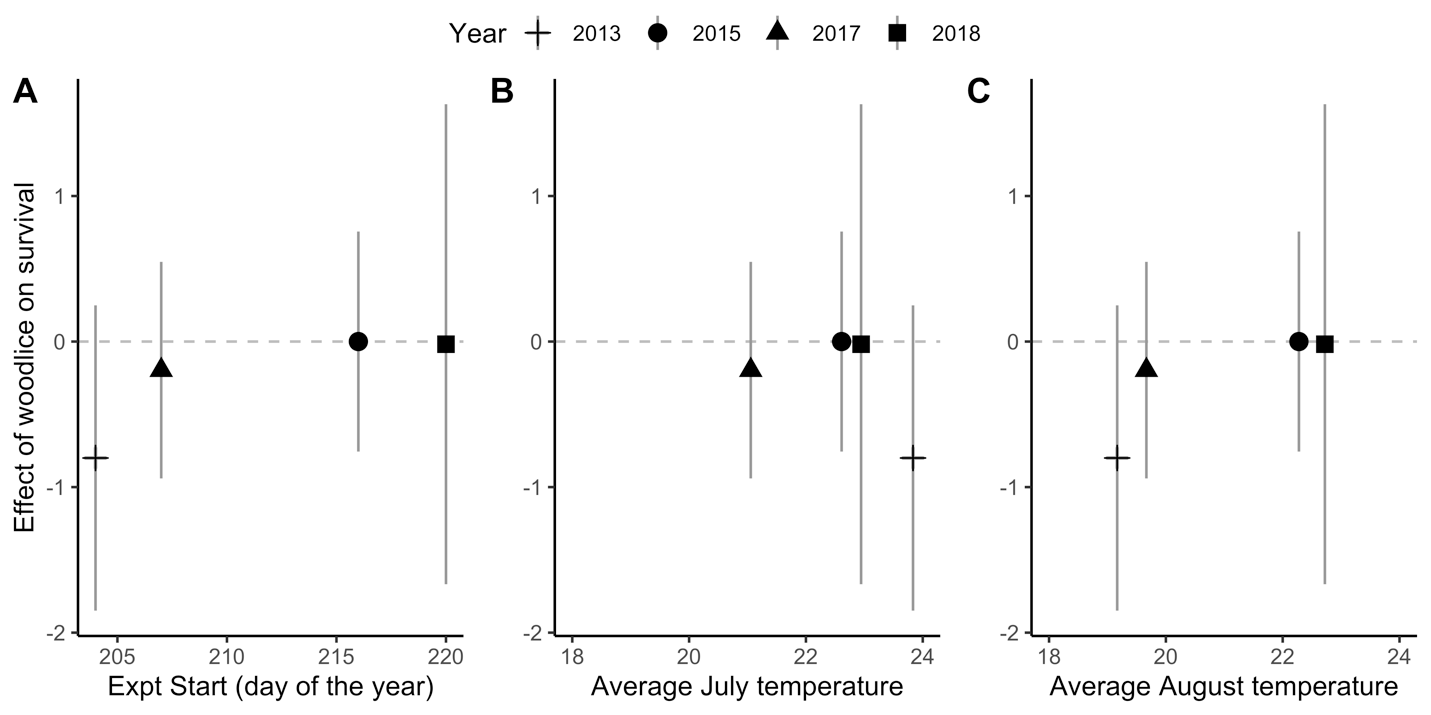


Figure S2: The effect of experiment (Expt) start date, average July temperature (°C), and average August temperature (°C) on the difference in grasshopper survival in cages with and without woodlice. Negative values indicated that woodlice reduced grasshopper survival. Error bars represent standard deviation, which is the square root of the sum of the variance in density in the woodlouse versus woodlouse-free cages.


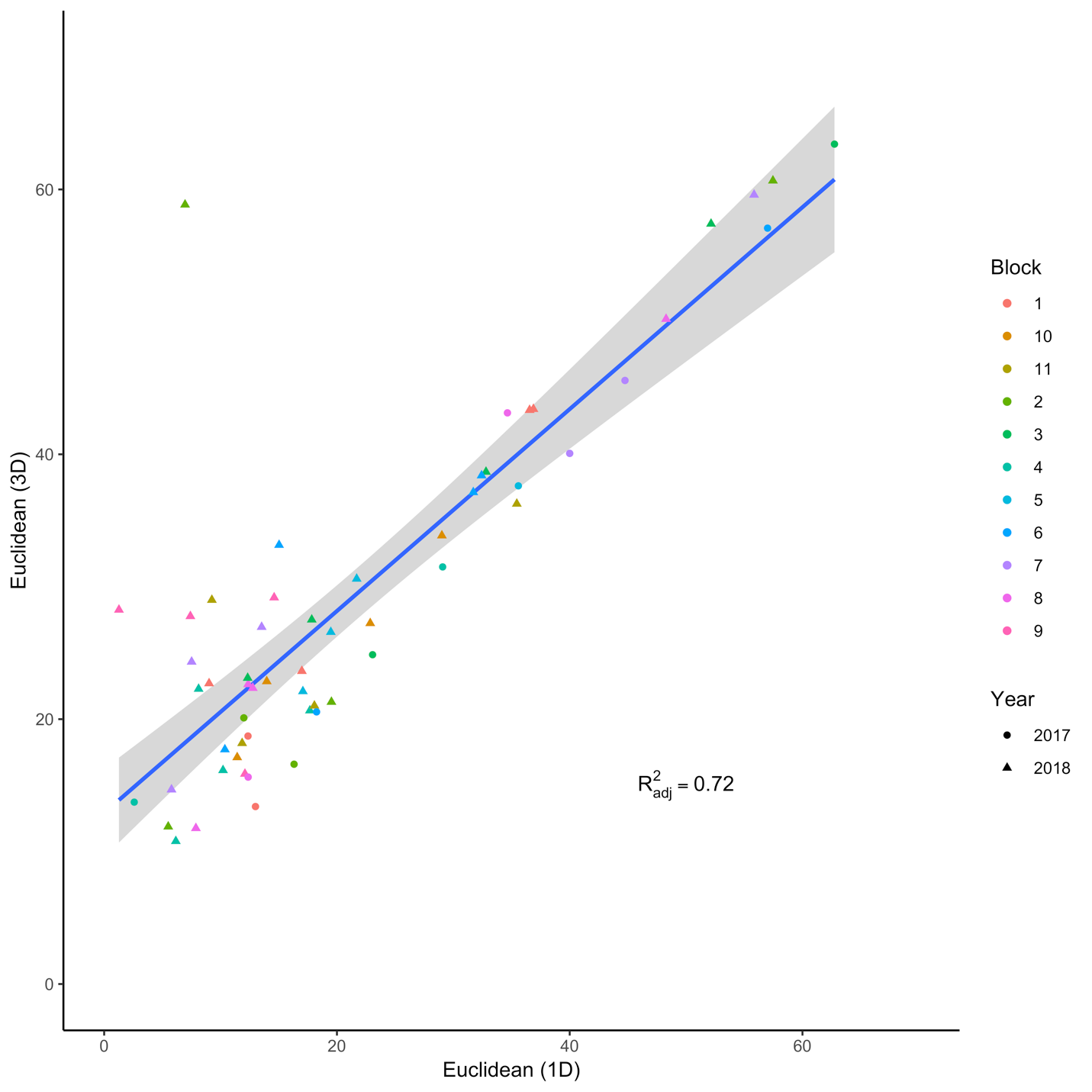


Figure S3: The average distance between grasshoppers and spiders in each cage measured in 1 dimension (height) and 3 dimensions. The difference in grasshopper and spider height was an accurate predictor of the difference in Euclidean distance between them. The outlier in the top left-hand corner is a cage where both grasshoppers and the spider spent significant time at the top of the cage in opposite corners.


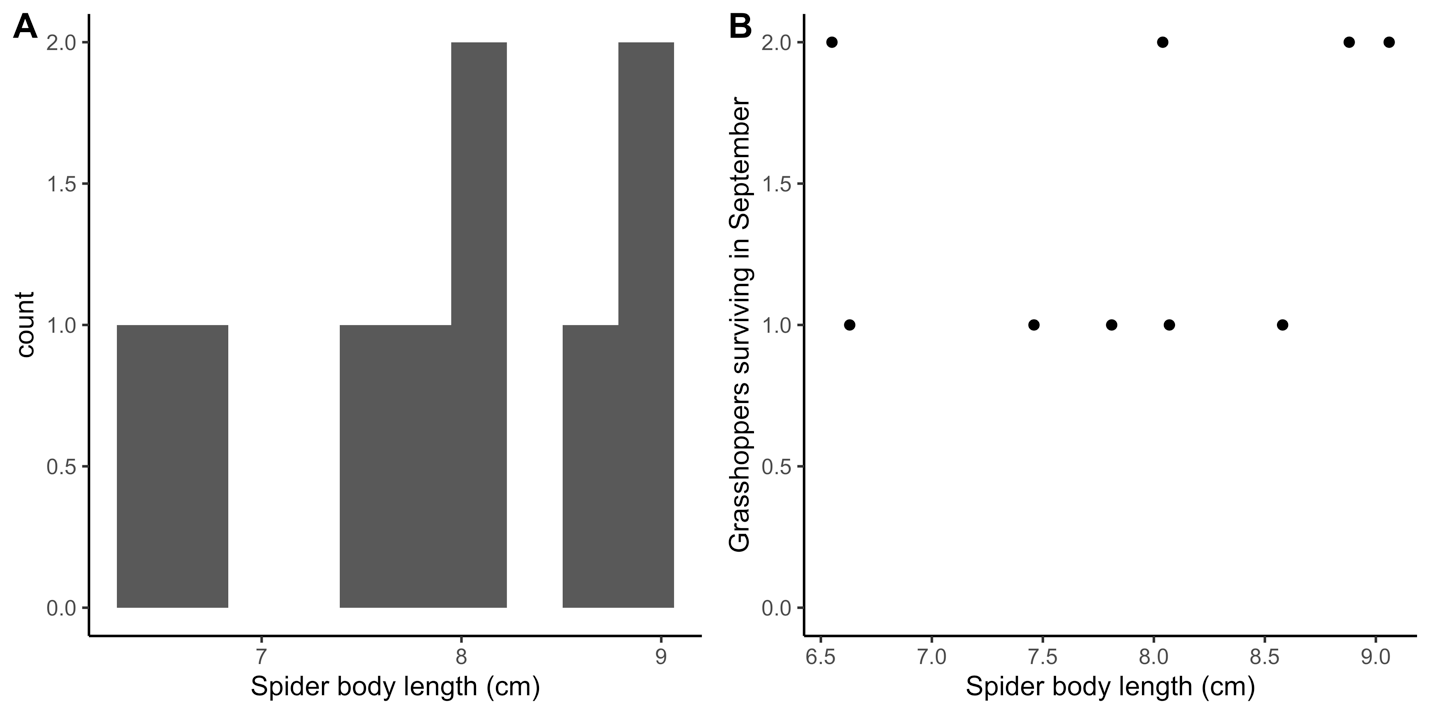


Figure S4: The distribution of spider body length used in the 2015 experiment (A). Spider body length did not predict grasshopper survival at the end of the experiment in 2015 (B).

**Section S2: Detailed statistical results**

Table S1: The model results for a Bayesian hierarchical model predicting spider (PIMI) and grasshopper (MEFE) height based on woodlouse treatment (SIG = woodlice) and temperature (TempW=warmed). The model has a nested effect of block within year. The equations are in the main text (Eqn 1). The model can be re-run in GreenBrownAnalysis.R: Section 1.1.

|  | **Estimate** | **Error** | **CI Lower-95%** | **CI Upper-95%** | **Effective Sample** | $\hat{\boldsymbol{R}}$ |
| --- | --- | --- | --- | --- | --- | --- |
| Group-Level Effects ~ Year (3) | | | | | | |
| sd(MEFE_Intercept) | 3.35 | 3.30 | 0.12 | 12.99 | 2017 | 1 |
| sd(PIMI_Intercept) | 13.03 | 10.67 | 0.88 | 39.87 | 2418 | 1 |
| Group-Level Effects ~ Year: Block (20) | | | | | | |
| sd(MEFE_Intercept) | 2.01 | 1.54 | 0.07 | 5.65 | 2411 | 1 |
| sd(PIMI_Intercept) | 3.51 | 2.76 | 0.17 | 10.29 | 4240 | 1 |
| Population-Level Effect | | | | | | |
| MEFE_Intercept | 68.68 | 3.03 | 62.47 | 74.66 | 2443 | 1 |
| PIMI_Intercept | 49.78 | 9.26 | 31.52 | 69.50 | 1889 | 1 |
| MEFE_SIG | -1.44 | 2.70 | -6.81 | 3.83 | 3959 | 1 |
| MEFE_TempW | -1.58 | 3.80 | -8.94 | 5.92 | 2957 | 1 |
| MEFE_SIG:TempW | -0.45 | 5.11 | -10.59 | 9.70 | 2559 | 1 |
| PIMI_SIG | 16.52 | 6.09 | 4.36 | 28.27 | 3960 | 1 |
| PIMI_TempW | -0.45 | 6.71 | -13.65 | 12.64 | 3109 | 1 |
| PIMI_SIG:TempW | -19.28 | 10.67 | -40.65 | 1.45 | 3553 | 1 |
| Family Specific Parameters | | | | | | |
| sigma_MEFE | 9.66 | 0.89 | 8.11 | 11.58 | 5331 | 1 |
| sigma_PIMI | 23.06 | 2.03 | 19.51 | 27.39 | 4303 | 1 |
| Residual Correlations | | | | | | |
| rescor(MEFE,PIMI) | -0.02 | 0.12 | -0.26 | 0.22 | 4466 | 1 |

Table S2: The model results for a Bayesian hierarchical model predicting spider (PIMI) and grasshopper (MEFE) height based on woodlouse treatment (SIG = woodlice) and temperature (TempW=warmed) were six cages that had incomplete data are removed from the analysis. The qualitative conclusions are identical to the model presented in Table S1. The model has a nested effect of block within year. The equations are in the main text (Eqn 1). The model can be re-run in GreenBrownAnalysis.R: Section 1.2.

|  | **Estimate** | **Error** | **CI Lower-95%** | **CI Upper-95%** | **Effective Sample** | $\hat{\boldsymbol{R}}$ |
| --- | --- | --- | --- | --- | --- | --- |
| Group-Level Effects ~ Year (3) | | | | | | |
| sd(MEFE_Intercept) | 3.45 | 3.46 | 0.11 | 12.85 | 2559 | 1 |
| sd(PIMI_Intercept) | 12.82 | 11.17 | 0.61 | 40.74 | 2226 | 1 |
| Group-Level Effects ~ Year: Block (20) | | | | | | |
| sd(MEFE_Intercept) | 2.24 | 1.65 | 0.08 | 6.12 | 2236 | 1 |
| sd(PIMI_Intercept) | 4.15 | 3.23 | 0.2 | 11.76 | 3811 | 1 |
| Population-Level Effect | | | | | | |
| MEFE_Intercept | 68.71 | 3.21 | 62.09 | 75.11 | 2607 | 1 |
| PIMI_Intercept | 48.55 | 9.14 | 30.69 | 67.81 | 2320 | 1 |
| MEFE_SIG | -1.39 | 2.82 | -6.83 | 4.00 | 5624 | 1 |
| MEFE_TempW | -2.01 | 4.32 | -10.52 | 6.72 | 4857 | 1 |
| MEFE_SIG:TempW | -0.04 | 6.04 | -12.05 | 11.85 | 4413 | 1 |
| PIMI_SIG | 17.48 | 6.31 | 5.18 | 29.56 | 7609 | 1 |
| PIMI_TempW | -4.90 | 6.31 | -18.58 | 8.22 | 5995 | 1 |
| PIMI_SIG:TempW | -17.17 | 12.00 | -41.01 | 6.83 | 5459 | 1 |
| Family Specific Parameters | | | | | | |
| sigma_MEFE | 10.04 | 0.96 | 8.37 | 12.10 | 6063 | 1 |
| sigma_PIMI | 23.51 | 2.25 | 19.55 | 28.31 | 6771 | 1 |
| Residual Correlations | | | | | | |
| rescor(MEFE,PIMI) | -0.02 | 0.13 | -0.27 | 0.23 | 6918 | 1 |

Table S3: The model results for a Bayesian hierarchical model predicting spider (PIMI) and grasshopper (MEFE) height based on woodlouse treatment (SIG = woodlice) and temperature (TempW=warmed) without temperature priors. The qualitative conclusions are identical to the model presented in Table S1. The model has a nested effect of block within year. The equations are in the main text (Eqn 1). The model can be re-run in GreenBrownAnalysis.R: Section 1.0.

|  | **Estimate** | **Error** | **CI Lower-95%** | **CI Upper-95%** | **Effective Sample** | $\hat{\boldsymbol{R}}$ |
| --- | --- | --- | --- | --- | --- | --- |
| Group-Level Effects ~ Year (3) | | | | | | |
| sd(MEFE_Intercept) | 3.45 | 3.49 | 0.12 | 12.77 | 1836 | 1 |
| sd(PIMI_Intercept) | 11.90 | 10.84 | 0.59 | 39.31 | 2313 | 1 |
| Group-Level Effects ~ Year: Block (20) | | | | | | |
| sd(MEFE_Intercept) | 2.03 | 1.51 | 0.07 | 5.55 | 2278 | 1 |
| sd(PIMI_Intercept) | 3.45 | 2.73 | 0.13 | 10.19 | 3582 | 1 |
| Population-Level Effect | | | | | | |
| MEFE_Intercept | 68.78 | 3.17 | 62.63 | 75.15 | 2118 | 1 |
| PIMI_Intercept | 46.82 | 8.79 | 29.27 | 65.20 | 2197 | 1 |
| MEFE_SIG | -1.44 | 2.67 | -6.69 | 3.67 | 5487 | 1 |
| MEFE_TempW | -1.70 | 3.77 | =9.15 | 5.78 | 4664 | 1 |
| MEFE_SIG:TempW | -0.38 | 5.04 | -10.28 | 9.80 | 4140 | 1 |
| PIMI_SIG | 19.19 | 6.26 | 6.55 | 31.48 | 4370 | 1 |
| PIMI_TempW | 11.04 | 9.03 | -6.37 | 28.90 | 3680 | 1 |
| PIMI_SIG:TempW | -29.40 | 11.94 | -52.68 | -5.38 | 3403 | 1 |
| Family Specific Parameters | | | | | | |
| sigma_MEFE | 9.64 | 0.89 | 8.09 | 11.59 | 6472 | 1 |
| sigma_PIMI | 22.92 | 2.08 | 19.20 | 27.40 | 6518 | 1 |
| Residual Correlations | | | | | | |
| rescor(MEFE,PIMI) | -0.02 | 0.12 | -0.26 | 0.21 | 7416 | 1 |

Table S4: The model results for a Bayesian hierarchical model predicting grasshopper survival based on predicted spider attack rate. The equations are presented in the main text (Eqn 2). The model can be rerun in GreenBrownAnalysis.R: Section 2.2.

|  | **Estimate** | **Error** | **CI Lower-95%** | **CI Upper-95%** | **Effective Sample** | $\hat{\boldsymbol{R}}$ |
| --- | --- | --- | --- | --- | --- | --- |
| Group-Level Effects ~ Year (3) | | | | | | |
| sd(Intercept) | 2.31 | 2.49 | 0.15 | 9.45 | 1180 | 1 |
| Group-Level Effects ~ Year: Block (20) | | | | | | |
| sd(Intercept) | 0.59 | 0.30 | 0.06 | 1.21 | 1416 | 1 |
| Population-Level Effect | | | | | | |
| Intercept | -0.22 | 1.69 | -3.60 | 3.16 | 1077 | 1 |
| Attack rate | -0.53 | 8.56 | -17.41 | 16.01 | 6156 | 1 |

**Section S3: Wolf spider analysis**

The wolf spider analysis was conducted separately from the main analysis, because we did not observe wolf spiders often enough to fit the multinomial model. Our observations confirmed that they primarily spend their time in or below the litter layer, since only 3 observations were recorded of which one was on top of the litter. We ran the following Bayesian model to test the effect of wolf spiders on grasshopper height.

| $L_{i}\sim Normal(\mu_{i}, \sigma)$  $\mu_{i}= \alpha+\alpha_{Block\left[ j \right]}+\beta_{GW}{GW}_{i}+\beta_{W}W_{i}$  $\alpha\sim T_{3}\left( 68, 13 \right)$  $\sigma\sim T_{3}\left( 0,10 \right)$  $\alpha_{Block\left[ j \right]}\sim T_{3}(0,10)$  $j=\left\{ 1,2,\ldots, 8 \right\}$ | (S1) |
| --- | --- |

The model (Eqn S1) sets the grasshopper and woodlouse treatment as the baseline and compares it with treatments containing only woodlice (“W”) and those containing woodlice and *G. gulosa* (“GW”). The number of replicates in these treatments were different. One cage containing *G. gulosa* and woodlice was set up, but no data were recorded because of an observer error. The grasshopper and woodlice cages were replicated more, because they were also set up as a possible comparison with the *P. mira* treatments.

Grasshoppers moved upwards in the canopy when wolf spiders were present (Figure S4A, Table S5). The grasshopper survival at the end of the summer was not affected by the presence of wolf spiders (Figure S4B).


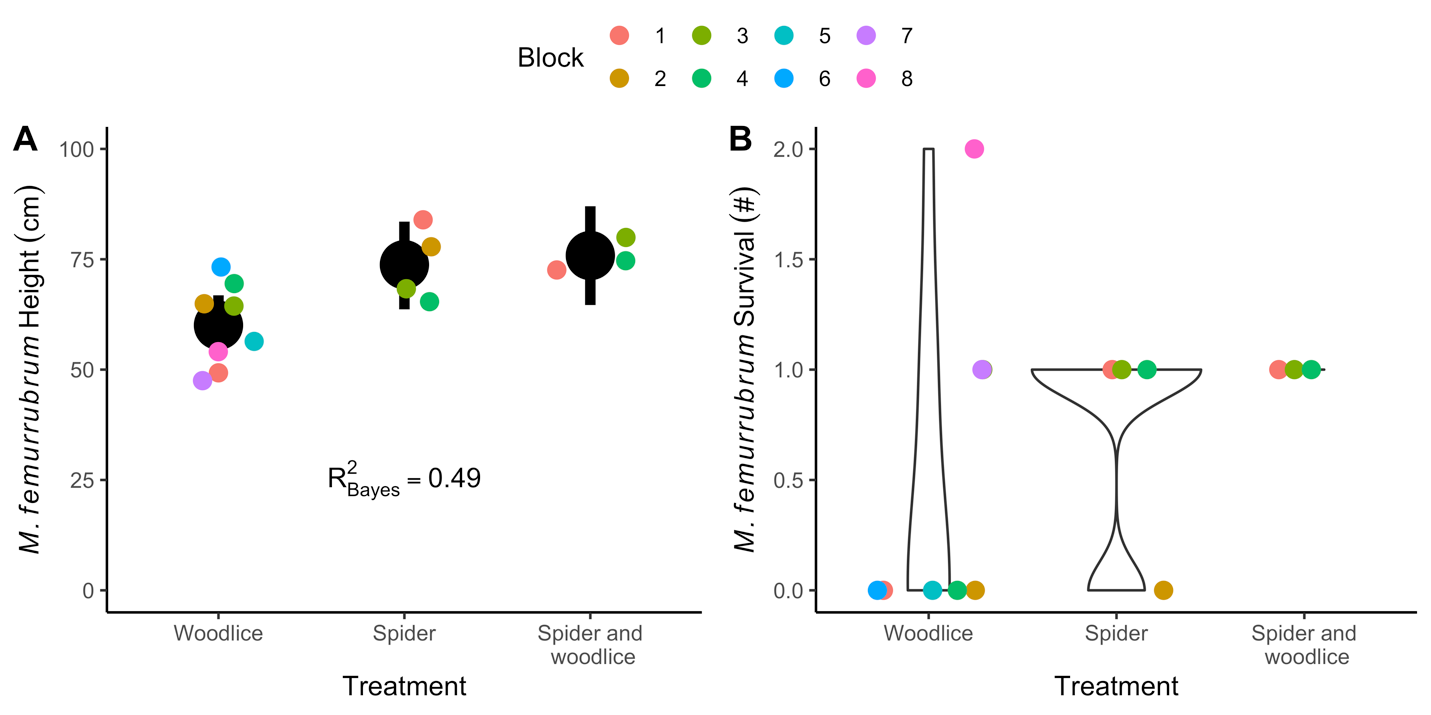


Figure S5: The effect of a sit-and-pursue spider *Gladicosa gulosa* on the height (A) and survival (B) of grasshoppers in the canopy. The presence of *G. gulosa* increased the height of the grasshoppers regardless of woodlouse presence, while grasshopper survival was unaffected (Table S3). These experiments were conducted in 2017. Note the difference in sample size. The woodlice only treatment had eight replicates, the spider only treatment had 4, and the combined treatment had three. The fourth combined cage was set up and stocked, but the data were not collected because of observer error.

Table S5: The model results for a Bayesian hierarchical model predicting grasshopper height based on the presence of woodlice and the sit-and-pursue spider *Gladicosa gulosa*. The baseline treatment (i.e. Intercept) was set to be cages with woodlice but no spiders. Note that the treatments had different sample sizes (Figure S4). The model can be rerun in GreenBrownAnalysis.R: Section 5.0.

|  | **Estimate** | **Error** | **CI Lower-95%** | **CI Upper-95%** | **Effective Sample** | $\hat{\boldsymbol{R}}$ |
| --- | --- | --- | --- | --- | --- | --- |
| Group-Level Effects ~ Block (8) | | | | | | |
| sd(Intercept) | 3.45 | 2.82 | 0.14 | 10.30 | 2393 | 1 |
| Population-Level Effect | | | | | | |
| Intercept (Woodlice) | 59.98 | 3.45 | 53.03 | 66.85 | 4218 | 1 |
| +*G. gulosa* & -woodlice | 13.72 | 5.74 | 2.19 | 24.94 | 4869 | 1 |
| +G. gulosa & +woodlice | 15.85 | 6.35 | 3.30 | 28.09 | 4492 | 1 |

**Section S4: Additional theoretical model results**

The encounter probabilities in all three models was based on the vertical habitat domains built for *P. mira*, *M. femurrubrum*, and *O. asellus*. These distributions were normally distributed for *P. mira* and *M. femurrubrum* (Figure S5). The distribution of *O. asellus* was built by taking observations from 2017—the only year isopods were observed—and fitting a gamma distribution to their heights (theoretical_model_analysis.R Section 1.2.2). The resulting distribution accurately reflected our observations that *O. asellus* does climb plants on rare occasions, but is most often observed in the bottom 5-cm where litter stacks up on the soil.

Our analysis of the signal detection theory model demonstrated that spiders should almost always attack a potential prey item regardless of whether they can correctly identify it as grasshopper prey or woodlouse non-prey. There are cases within parameter space where this is not the best strategy. They occur if spiders are not able to distinguish grasshoppers and woodlice very well and the risk of potential prey items escaping is high (Figure S6A). Learning to distinguish grasshoppers and woodlice should be valuable when there is no time to carefully observe them and a prey item is likely to escape quickly. Interestingly, the spider’s ability to successfully catch the grasshoppers they attack has no qualitative impact on the model, unless spiders are terrible at catching grasshoppers (success rate = 10%, Figure S6B). Finally, spiders do employ a more conservative strategy if the cost of attacking a woodlouse is raised three orders of magnitude above what we estimated as the metabolic cost (Figure S6C).

The same qualitative dynamics appear when spiders are forced to always attack prey items in the net energy gain model (Figure S7). Woodlice only impact net energy gain when spiders are low in the canopy and attack cost is high (Figure S7A) and increasing attack success rate increases the expected energy gain but doesn’t change the marginal effect of woodlice (Figure S7B).

In individual-based models, the spiders move upwards in the canopy because of a bias towards moving lower in the canopy near woodlice. The shift occurs regardless of how the spiders respond to grasshoppers (c.f. Figure 2B, Figure S8A). However, there is no net change in spider height when the spiders have a high movement rate independent of encountering other animals (Figure S8B). This would be the case for an active hunting species.


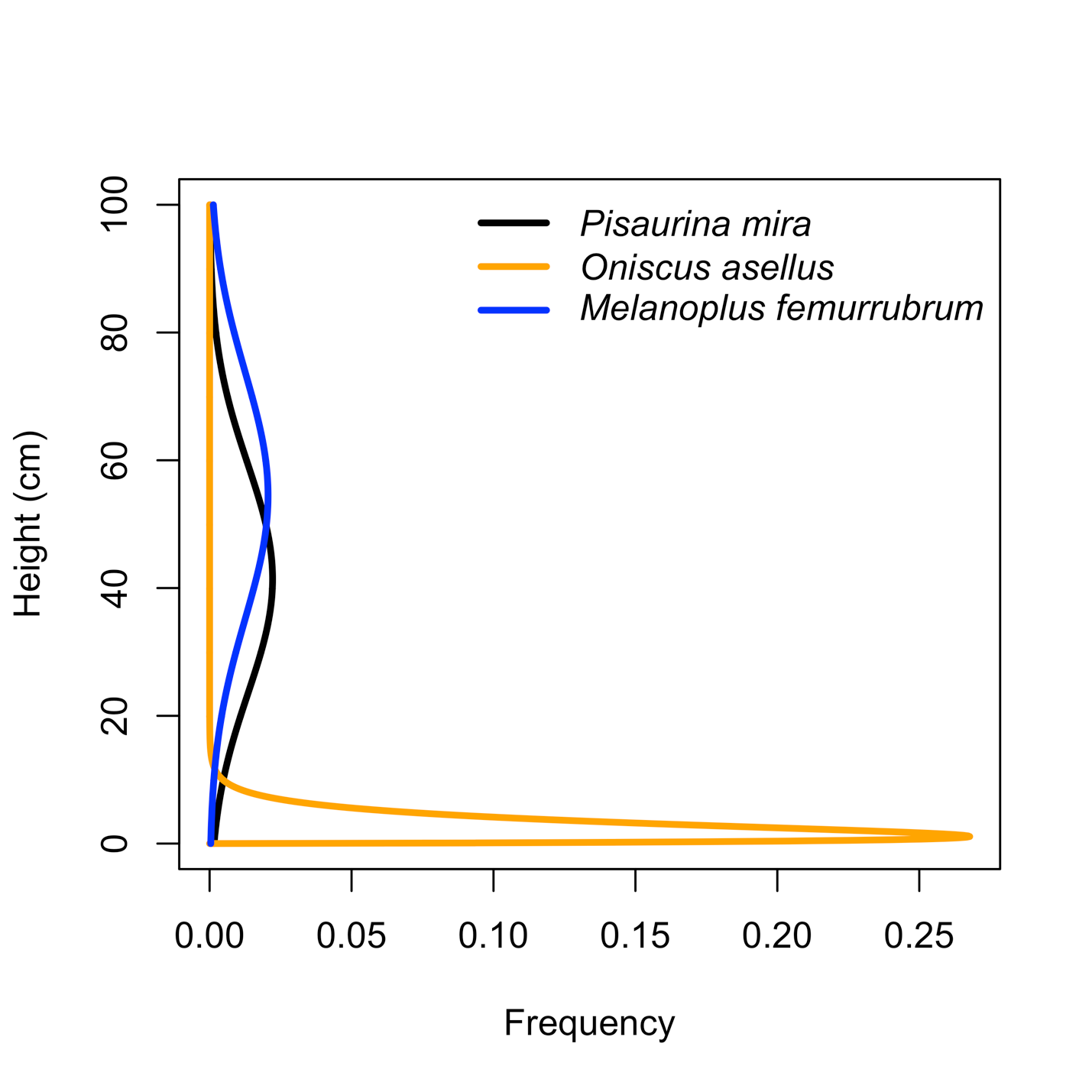


Figure S6: The height distribution for the three species included in our model under ambient conditions. The spider *P. mira* preys upon the grasshopper *M. femurrubrum* and may mistakenly attack the woodlouse *O. asellus*. The spider and grasshopper heights are normally distributed. The woodlouse height is gamma distributed. The spider’s average height is modified to find the optimal distribution given the opportunities to attack grasshoppers and avoid woodlice and high temperatures. You can recreate this figure in theoretical_model_analysis.R: Section 1.4.


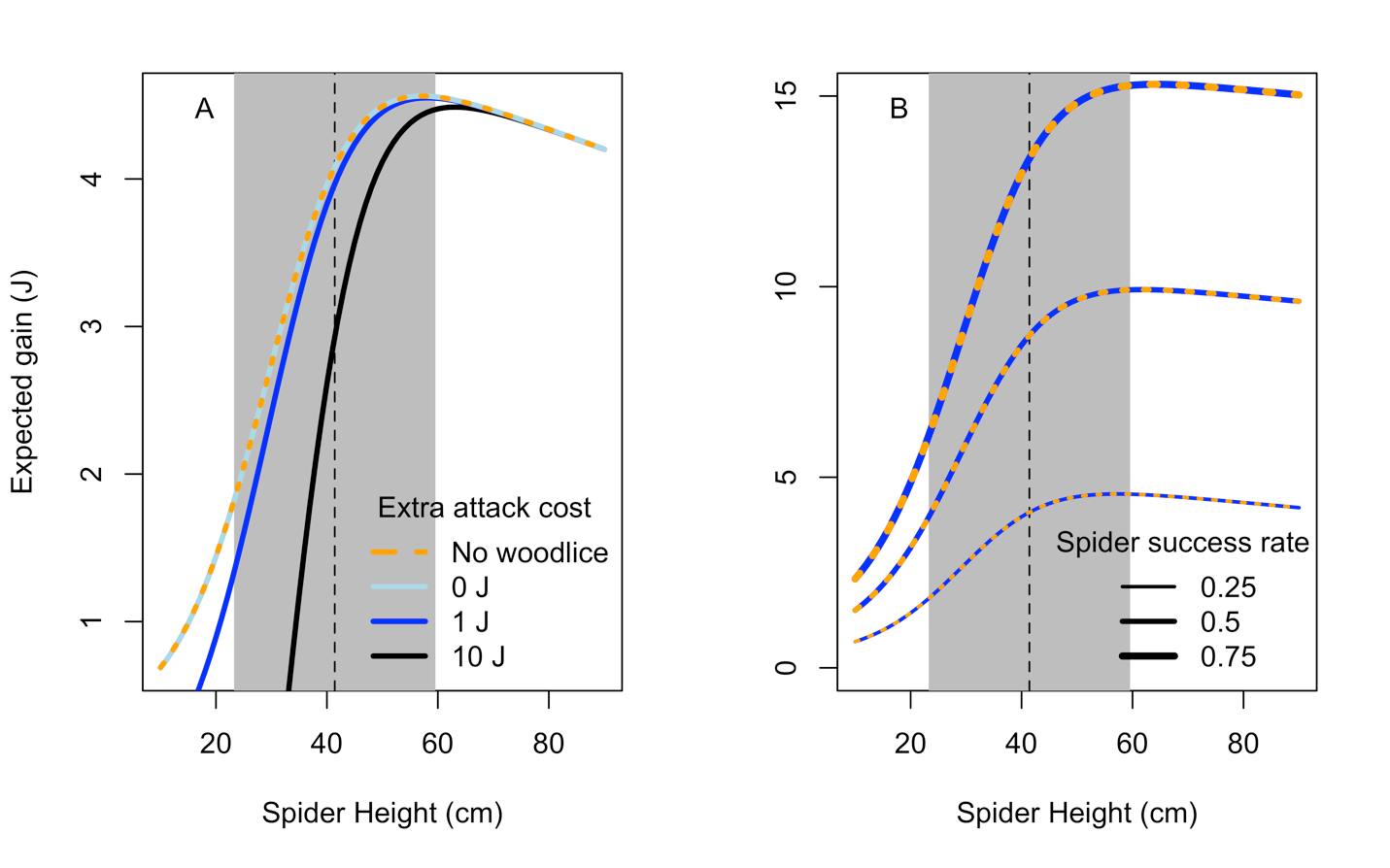
 Figure S7: The expected energy gain based on spider height, the presence of woodlice, and two parameter modifications in the net energy gain model. Attacking a woodlouse needs to be at least three orders of magnitude costlier (> 1 J) to have a notable cost on spiders when they are low in the canopy near woodlice (A). Increasing spider attack success rate on grasshoppers increases their expected energy gain, but does not change the effect of woodlice on net energy gain (B). You can recreate this figure in theoretical_model_analysis.R: Section 3.


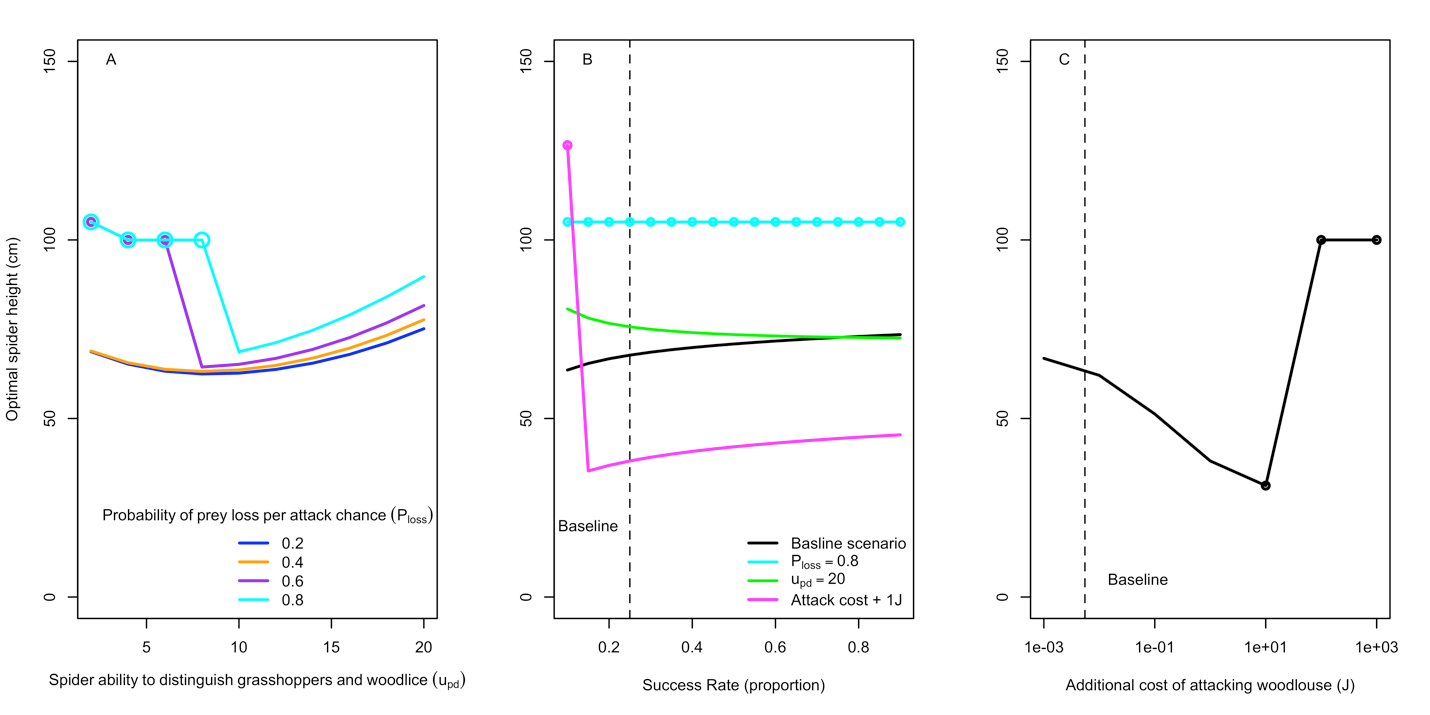


Figure S8: The results of our analysis using signal detection theory and the spatial overlap of woodlice, grasshoppers, and spiders. Lines trace the optimal spider height. In panel A, the optimal spider height varies with the spider’s ability to distinguish woodlice and grasshoppers (x-axis) and the probability that the prey item will escape before an attack can be attempted (colors). In panel B, the optimal spider height varies based on the spider attack success rate (x-axis) and changes in probability of loss, distinction ability, and the cost of attacking woodlice. In panel C, the optimal spider height varies based on an additional cost of attacking woodlice beyond the cost of active respiration. Circles indicate cases where spiders should learn whether potential prey is edible to reach an optimal strategy. Overall, changes to the baseline parameters do not change the qualitative model behavior unless the spiders ability to distinguish between prey and non-prey is low and probability of loss is high or attack cost is elevated. You can recreate this figure in theoretical_model_analysis.R: Section 2.2.


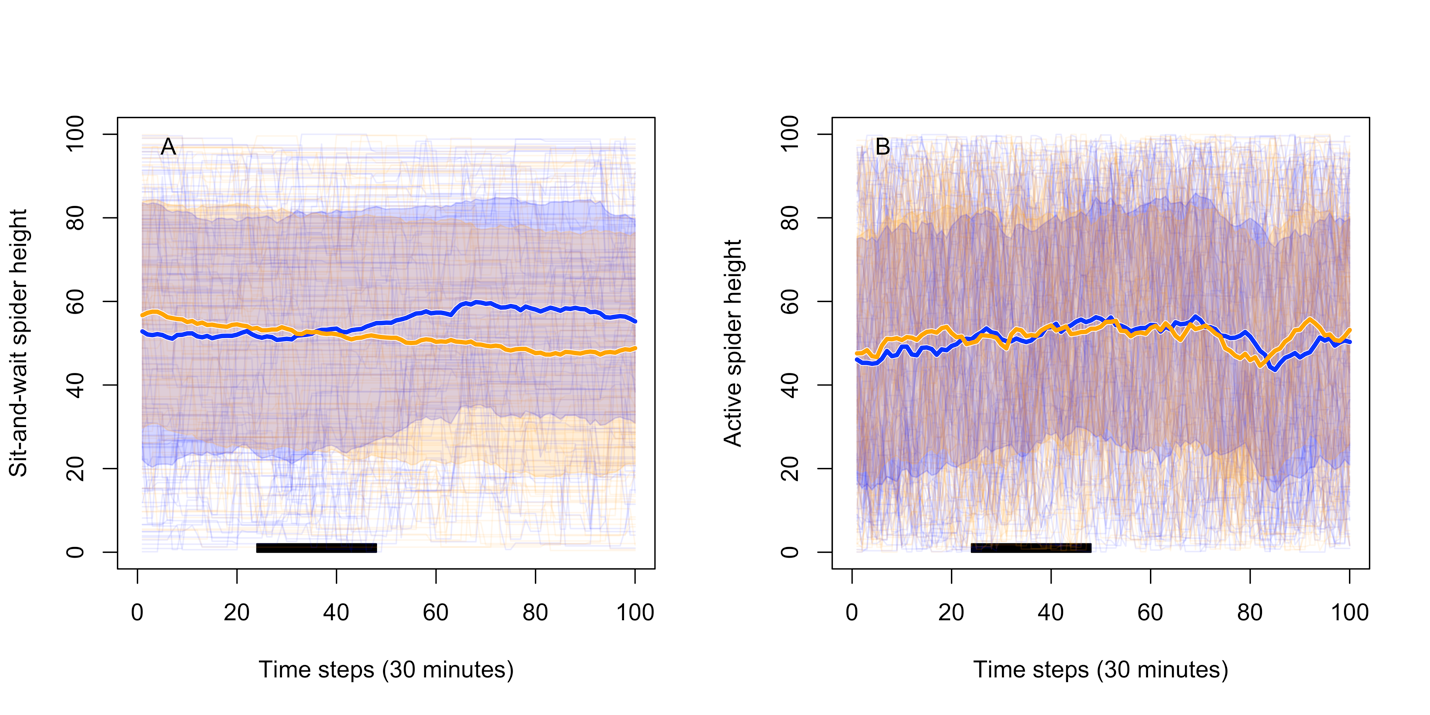
Figure S9: The results of an individual-based simulation model where the spider moves after encountering either woodlice or grasshoppers unless they successfully capture a grasshopper. Panel A shows a simulation for a nursery web spider with a probability of unstimulated movement of 0.1. These results are the same as in the model presented in the main text. Panel B shows a simulation for a more active hunting spider (e.g. *Phidippus clarus*), which is more likely to move without stimulus (probability = 0.8). These results show no difference between the treatments. You can recreate this figure in theoretical_model_analysis.R: Section 4.
